# Association of Remote History of Venous Thrombosis With Risk of Venous Thrombosis After Age 70 Years

**DOI:** 10.1001/jamanetworkopen.2022.4205

**Published:** 2022-03-25

**Authors:** Huijie Wang, Mary Cushman, Frits R. Rosendaal, Astrid van Hylckama Vlieg

**Affiliations:** 1 Leiden University Medical Center, Department of Clinical Epidemiology, Leiden, the Netherlands; 2 Department of Medicine, Larner College of Medicine at the University of Vermont, Burlington

## Abstract

**Importance:**

Previous venous thrombosis (VT) is associated with risk of future VT, but quantification of risk over the life course is poorly understood. More information is needed for clinicians to understand the association of remote history of VT with the risk of VT in older patients.

**Objective:**

To assess the association between a remote history of VT and the development of VT in older age.

**Design, Setting, and Participants:**

The Age and Thrombosis, Acquired and Genetic Risk Factors in the Elderly case-control study enrolled patients 70 years and older with VT and control individuals 70 years and older without VT between June 2008 and August 2011. The Age and Thrombosis, Acquired and Genetic Risk Factors in the Elderly study is a 2-center, population-based case-control study that was conducted in Burlington, Vermont, in the US and in Leiden, the Netherlands. Consecutively diagnosed patients with an objectively proven episode of VT (deep vein thrombosis of the leg or pulmonary embolism) were included. Control individuals were identified in the same geographic areas as the patients and were randomly selected. Data were analyzed between May 2021 and October 2021.

**Exposures:**

Self-reported remote VT (occurring >10 years before to enrollment).

**Main Outcomes and Measures:**

The main outcome was the risk of VT at older age. The association of self-reported history of remote VT with VT at older age was assessed by calculating odds ratios (ORs) as estimates of relative risk with 95% CIs.

**Results:**

A total of 460 patients with VT and 456 control participants were included. There were slightly more women than men in both groups (60.2% of patients [n = 277] were women and 52.4% of control participants [n = 239] were women), and the mean (range) age of patients was 78.7 (70.0-100.9) years, which was similar to the control participants. Compared with individuals without remote VT, those with a remote history of VT had an increased risk of VT (OR, 2.54; 95% CI, 1.56-4.13). The crude risk estimate was robust to adjustment and time since remote VT, that is, individuals with a VT 10 to 30 years ago (OR, 2.74; 95% CI, 1.34-5.57) and those with a VT more than 30 years ago (OR, 2.42; 95% CI, 1.21-4.84) had a an increased risk of VT. The population-attributable fraction of a remote history of VT was 7.7%.

**Conclusions and Relevance:**

In this study, a remote history of VT was associated with risk of VT in older individuals. This quantification could assist clinicians in advising patients on VT prevention.

## Key Points

**Question:**

Is remote history of venous thrombosis (VT) associated with an increased risk of VT in older adults?

**Findings:**

In this case-control study of 460 patients with VT and 456 control participants, those with a remote history of VT had a higher risk of VT compared with individuals without a history of VT.

**Meaning:**

Findings of this study suggest that a remote history of VT may be associated with the risk of VT in older adults, and this quantification could assist clinicians in advising patients on VT prevention in risk settings, such as surgery.

## Teaser

This case-control study evaluates the association between a remote history of venous thrombosis and later risk of venous thrombosis in older adults.

## Introduction

Venous thrombosis (VT) is the third most common cardiovascular disease, after myocardial infarction and stroke. Venous thrombosis includes deep vein thrombosis (DVT) and pulmonary embolism (PE). The risk of VT increases with age^1^; 60% of all events occur after the age of 60 years. The incidence of VT is less than 5 per 100 000 individuals per year in people younger than 15 years but is approximately 500 patients per 100 000 individuals per year at the age of 80 years.^2^

Venous thrombosis has a strong tendency to recur, with a recurrence rate of approximately 5% per year,^3^ and VT may occur even in the presence of adequate therapy.^4^ Few studies that focus on the risk of recurrence of VT have information over decades of follow-up. Among the studies with longer duration of follow-up, the cumulative recurrence was reported to be approximately 10% after 1 to 2 years and increased to 44% to 56% after 20 years since the initial event.^5,6^ A 2019 meta-analysis^7^ showed that the cumulative recurrence risk of VT was 10% in the first year after anticoagulant treatment, 16% at 2 years, 25% at 5 years, and 36% at 10 years after a first VT.^7^ The recurrence rate is not stable over time. In the available studies with a follow-up time of more than 10 years, the recurrence rate was high during the first year after the first VT (ie, 7.8 per 100 person-years) after which the rate attenuated in the follow-up period (1-18 years).^5^

Considering that VT is common, that aging is the most important risk factor for VT, that the risk of VT diminishes over time, and that the longest follow-up available in most observational studies after VT is approximately 10 years, more information is needed for clinicians to understand the association of remote history of VT with risk of future VT in older individuals.

To our knowledge, there are no prediction models for the risk of VT specifically in older adults. The aim of this study was to assess whether a remote history of VT (ie, a VT more than 10 years ago) was associated with the risk of VT in older patients.

## Methods

### Study Design and Participation

The Age and Thrombosis, Acquired and Genetic Risk Factors in the Elderly (AT-AGE) study is a 2-center, population-based case-control study carried out in Leiden, the Netherlands and Burlington, Vermont, in the US. The design has been described previously.^6^ In brief, consecutive patients were enrolled from June 1, 2008, to August 20, 2011, in Leiden and from December 1, 2008, to July 20, 2011, in Vermont. All participants were 70 years or older. Patients with an objectively diagnosed, proven episode of VT, that is, deep vein thrombosis (DVT) of the leg or a pulmonary embolism (PE [with or without DVT]) were included. These consecutively diagnosed patients were recruited on the basis of imaging test results that showed evidence of the conditions. Diagnostic tests included compression ultrasonography, Doppler ultrasonography, impedance plethysmography, and contrast venography for diagnosis of DVT as well as perfusion and ventilation lung scanning, spiral computer tomography, and pulmonary angiography for diagnosis of PE. Neither patients nor control participants were excluded owing to the use of anticoagulation drugs. In Leiden, patients were identified from 2 anticoagulation clinics in a defined area in the western part of the Netherlands. In Vermont, sequential patients were identified through testing in all imaging centers in the area. Control participants were identified in the same geographic areas as the patients and were randomly selected from 5 primary care practices in Leiden and 4 practices in Vermont. Both patients and control participants were invited by letter, followed by a telephone call to discuss participation. Exclusion criteria were a diagnosis of cancer, receipt of chemotherapy or radiotherapy 6 months prior to study entry, and a recent episode of VT (within the last 10 years). Participants with severe psychiatric or cognitive disorder, as judged during telephone contact, were also excluded. In total, of 1187 patients and 723 control participants identified, 498 patients and 92 control individuals were excluded, and 216 eligible patients and 170 control participants refused to participate owing to time constraints or illness. This left 473 patients and 461 control participants who were interviewed. Owing to 13 patients and 5 control participants having incomplete interview data, 460 patients and 456 control participants were included in the study.

All participants were visited at their homes by a trained research assistant for an interview and a venipuncture. All participants provided written informed consent. The study was approved by the medical ethical committee of the Leiden University Medical Center and by the committee of human research of the University of Vermont. This study followed the Strengthening the Reporting of Observational Studies in Epidemiology (STROBE) reporting guideline. Race reporting was based on self-reported data; we collected data on the country of birth of participants and their parents and did not collect data specifically on ethnicity.

### Data Collection and Definitions

During the home visit, an interview was conducted to ascertain details about the VT (for patients), medical history, family history of VT, and lifestyle habits. Weight, height, and blood pressure were measured. Furthermore, all participants provided information regarding the exposure, that is, a remote VT history. Remote VT history was defined as self-reported VT occurring more than 10 years prior to the index date. A venipuncture was performed by a trained research assistant using standard methods. The index date was defined as the date of diagnosis of the current thrombosis for the patients and the date of the home visit for the control participants.

Venous thrombosis was classified as provoked if it occurred after hospitalization (including recent surgery), fracture, plaster cast, splint, minor injuries of the lower extremities (eg, a sprained ankle or contusion of the lower leg), or transient immobilization at home for at least 4 successive days in the 3 months before the index date.

### Statistical Analysis

Data were analyzed between May and October 2021. First, descriptive statistics for all participants were presented as frequencies and percentages for categorical variables and as means and ranges for continuous variables. Second, we calculated the crude relative risk of VT associated with a remote history of VT (ie, any VT >10 years prior to the index date) compared with not having a history of VT to assess the crude value of a remote history of VT on the development of VT after age 70 years. This value was calculated both overall and separately for the risk of DVT and PE and for provoked and unprovoked events. Third, participants who reported a remote VT were divided into 2 groups based on the time between the prior and the current VT, that is, a prior event 10 to 30 years prior to the index date and a prior event more than 30 years prior to the index date. Fourth, we adjusted the risk estimates for available VT risk factors. Two models were used, one with study center and the standard available risk factors for a VT at older age (age, sex, body mass index [BMI, calculated as weight in kilograms divided by height in meters squared], and family history of VT) and one also including genetic markers of VT, factor V Leiden, a variant in the prothrombin gene G20210 (factor II A^20210^), and non–O blood groups.

The relative risk of VT was estimated by calculating odds ratios (ORs) with corresponding 95% CIs, using multivariable logistic regression models. This study is a case-control study sampled from a dynamic population. In this study setting, the OR is a perfect estimation of the rate ratio (relative risk) and can be interpreted as such.^8^

The population-attributable risk (PAR) was calculated as the proportion of cases exposed to the risk factor of interest multiplied by (OR minus 1 divided by OR). The PAR indicates the proportion of the total incidence of VT in individuals 70 years and older who were eligible for this study that can be attributed to a remote history of VT. In addition, to determine whether the association of remote VT with the risk of VT was consistent across key groups, we assessed VT risk after dichotomization by age (dichotomized at the mean age of the whole study population, 78.1 years), sex, and BMI (dichotomized at 25). All analyses were performed in SPSS, version 23.0 for Windows (SPSS Inc).

## Results

In total, 460 patients and 456 control participants were reported in our study (Table 1). The mean (range) age of patients was 78.7 (70.0-100.9) years, which was similar to that of the control participants (mean age, 77.5; range, 70.3-96.3 years). There were slightly more women than men in both groups (277 [60.2%] vs 183 [39.8%] in the patient group and 239 [52.4%] vs 217 [47.6%] in the control group). Most study participants (412 [89.6%] in the patient group and 379 [83.1%] in the control group) self-reported White race and ethnicity. Body mass index was similar in both groups. Of the patients, 196 (42.6%) had DVT only, whereas 263 (57.2%) were diagnosed as having PE (with or without DVT). Approximately half of patients had provoked VT. Remote history of VT was present in 59 patients (12.8%) and 25 control participants (5.5%). All provoking VT risk factors and genetic risk factors were more common in patients than in control participants.

**Table 1.** Population Characteristics

Participant characteristics	No. (%)	
	Patients (n = 460)^a^	Control participants (n = 456)^b^
Age, mean (range), y	78.7 (70.0-100.9)	77.5 (70.3-96.3)
Sex		
Female	277 (60.2)	239 (52.4)
Male	183 (39.8)	217 (47.6)
White race^c^	412 (89.6)	379 (83.1)
BMI, mean (range)	27.3 (14.4-52.0)	27.0 (17.0-49.7)
VT >10 y ago	59 (12.8)	25 (5.5)
Time since prior VT, mean (range), y	31.2 (11.0-56.0)	33.6 (14.0-56.0)
Type of current VT		
DVT	196 (42.6)	NA
PE with or without DVT	263 (57.2)	NA
Provoked VT	213 (46.3)	NA
Unprovoked VT	235 (51.1)	NA
Provoking factors		
Hospital admission	143 (31.1)	31 (6.8)
Surgery	79 (17.2)	16 (3.5)
Fracture	30 (6.5)	3 (0.7)
Plaster cast	22 (4.8)	4 (8.8)
Immobilization	41 (8.9)	8 (1.8)
Minor injury	54 (11.7)	35 (7.7)
Genetic factors		
Factor V Leiden	47 (10.2)	19 (4.2)
Variant in the prothrombin gene (factor II A^20210^)	11 (2.4)	8 (1.8)
Non–O blood groups	278 (60.4)	247 (54.1)
Family history of VT	124 (30.0)	61 (13.4)

Abbreviations: BMI, body mass index (calculated as weight in kilograms divided by height in meters squared); DVT, deep vein thrombosis; NA, not applicable; PE, pulmonary embolism; VT, venous thrombosis.

^a^ Missing in patients: 24 for BMI; 4 for mean time since prior VT; 11 for race and ethnicity; 12 for immobilization; 7 for minor injury; 1 for DVT/PE with or without DVT; 12 for provoked or unprovoked VT; 8 for factor V Leiden; 8 for factor II A^20210^; and 28 for non–O blood group.

^b^ Missing in control participants: 10 for BMI; 2 for mean time since prior VT; 8 for ethnicity; 1 for fracture; 2 for immobilization; 1 for minor injury; 6 for factor V Leiden; 4 for factor II A^20210^; 16 for non–O blood groups; and 1 for family history of VT.

^c^ Race reporting was based on self-reported data. Data were collected on the country of birth of the participants and their parents; data on other races and ethnicities were not collected.

Table 2 shows the risk of VT associated with a remote history of VT (crude estimate). Compared with individuals without a history of VT, the risk of VT in those with a remote history of VT was 2.54 (95% CI, 1.56-4.13). The PAR of a remote history of VT was 7.7%. The risk of VT was similar for those with a history of VT that occurred 10 to 30 years before the index date (OR, 2.74; 95% CI, 1.34-5.57) and those with a history that occurred more than 30 years before the index date (OR, 2.42; 95% CI, 1.21-4.84). The risk of VT associated with a remote history of VT was only mildly attenuated after adjustment for standard available risk factors (OR, 2.37; 95% CI, 1.39-4.04) and after further adjustment for genetic factors (OR, 2.21; 95% CI, 1.26-3.86) (Table 2).

**Table 2.** The Risk of VT Associated With a Remote History of VT

VT	Patients, No. (n = 460)	Control participants, No. (n = 456)	OR (95% CI)		
			Crude	Adjusted^a^	Adjusted^b^
No prior	401	431	1 [Reference]	1 [Reference]	1 [Reference]
All remote	59	25	2.54 (1.56-4.13)	2.37 (1.39-4.04)	2.21 (1.26-3.86)
Remote					
10-30 y ago^c^	28	11	2.74 (1.34-5.57)	2.54 (1.19-5.41)	2.05 (0.93-4.51)
>30 y ago^c^	27	12	2.42 (1.21-4.84)	2.05 (0.96-4.38)	2.21 (0.99-4.94)

Abbreviations: OR, odds ratio; VT, venous thrombosis.

^a^ Adjusted for age, sex, body mass index (calculated as weight in kilograms divided by height in meters squared), study center, and family history of VT (standard factors).

^b^ Adjusted for age, sex, body mass index, study center, family history of VT, factor V Leiden, variant in the prothrombin gene (factor II A^20210^) and non–O blood groups (extensive factors).

^c^ Exact time between former VT and index date is missing for 4 patients and 2 control participants.

Table 3 shows the risk of DVT, PE with or without DVT, provoked, and unprovoked events separately. The risk of VT at older age was higher for those with DVT (OR, 3.12; 95% CI, 1.78-5.46) than for those with PE with or without DVT (OR, 2.05; 95% CI, 1.17-3.60). The risk estimates were also higher for unprovoked (OR, 3.54; 95% CI, 2.09-5.99) than for provoked events (OR, 1.50; 95% CI, 0.79-2.83). Similar risk patterns were seen after adjustment for standard available risk factors and after further adjustment for genetic risk factors (Table 3).

**Table 3.** The Risk of DVT, PE With or Without DVT, and Provoked and Unprovoked VT Associated With a Remote History of VT

Variable	Patients, No. (n = 460)	Control participants, No. (n = 456)	OR (95% CI)			
			DVT	PE with or without DVT	Provoked	Unprovoked
Crude, VT						
No prior	401	431	1 [Reference]	1 [Reference]	1 [Reference]	1 [Reference]
All remote	59	25	3.12 (1.78-5.46)	2.05 (1.17-3.60)	1.50 (0.79-2.83)	3.54 (2.09-5.99)
Remote						
10-30 y ago^a^	28	11	3.07 (1.35-6.99)	2.33 (1.04-5.22)	2.00 (0.84-4.79)	3.22 (1.47-7.06)
>30 y ago^a^	27	12	2.81 (1.26-6.29)	2.14 (0.97-4.70)	1.28 (0.50-3.31)	3.68 (1.77-7.69)
Standard factors, VT^b^						
No prior	401	431	1 [Reference]	1 [Reference]	1 [Reference]	1 [Reference]
All remote	59	25	2.83 (1.52-5.27)	1.93 (1.03-3.60)	1.23 (0.59-2.53)	3.37 (1.89-6.02)
Remote						
10-30 y ago^a^	28	11	2.60 (1.08-6.23)	2.30 (0.94-5.58)	1.87 (0.73-4.80)	2.82 (1.21-6.57)
>30 y ago^a^	27	12	2.44 (0.98-6.09)	1.76 (0.74-4.18)	0.78 (0.25-2.42)	3.42 (1.53-7.64)
Genetic factors, VT^c^						
No prior	401	431	1 [Reference]	1 [Reference]	1 [Reference]	1 [Reference]
All remote	59	25	2.43 (1.24-4.76)	1.95 (1.02-3.72)	1.21 (0.57-2.58)	3.00 (1.62-5.53)
Remote						
10-30 y ago^a^	28	11	1.71 (0.64-4.52)	2.12 (0.86-5.25)	1.66 (0.62-4.45)	1.96 (0.80-4.84)
>30 y ago^a^	27	12	2.63 (0.98-7.03)	1.93 (0.78-4.79)	0.88 (0.27-2.84)	3.65 (1.55-8.59)

Abbreviations: DVT, deep vein thrombosis; OR, odds ratio; PE, pulmonary embolism; VT, venous thrombosis.

^a^ Exact time between former VT and index date is missing for 4 patients and 2 control participants.

^b^ Adjusted for age, sex, body mass index (calculated as weight in kilograms divided by height in meters squared), study center, and family history of VT (standard factors).

^c^ Adjusted for age, sex, body mass index, study center, family history of VT, factor V Leiden, variant in the prothrombin gene (factor II A^20210^), and non–O blood groups (extensive factors).

Table 4 shows the risk of VT associated with a remote history of VT in subgroups after dichotomization on the mean age at the index date (78.1 years), BMI, or sex. The risk of VT associated with a remote history of VT was increased in all subgroups; however, point estimates were higher in participants 78.1 years or younger compared with older participants, in those with a BMI of 25 or less compared with a BMI greater than 25, and in men compared with women. For example, compared with participants without a previous VT, participants with remote VT who were 78.1 years and younger had an increased risk of VT (OR, 2.83; 95% CI, 1.47-5.47); those older than 78.1 years with a remote VT also had an increased risk of VT (OR, 2.20; 95% CI, 1.06-4.54). This increased risk remained after full adjustment and across categories of time since VT. We performed subgroup analysis merely to assess the robustness of the estimates and for descriptive purposes, not to find important differences between groups. Therefore, we did not perform formal interaction testing.

**Table 4.** Risk of VT Associated With a Remote History of VT After Statification by Age, Sex, and BMI

Subgroup	Patients, No. (n = 460)	Control participants, No. (n = 456)	OR (95% CI)		
			Crude	Adjusted^a^	Adjusted^b^
Age at index ≤78.1 y^c^					
No prior VT	204	261	1 [Reference]	1 [Reference]	1 [Reference]
All remote VT	31	14	2.83 (1.47-5.47)	2.83 (1.39-5.78)	2.69 (1.25-5.76)
Remote VT					
10-30 y ago^d^	10	5	2.56 (0.86-7.60)	2.60 (0.83-8.20)	1.75 (0.50-6.17)
30 y ago^d^	19	8	3.04 (1.30-7.08)	2.95 (1.16-7.52)	3.48 (1.27-9.54)
Age at index >78.1 y^c^					
No prior VT	197	170	1 [Reference]	1 [Reference]	1 [Reference]
All remote VT	28	11	2.20 (1.06-4.54)	2.03 (0.90-4.59)	1.94 (0.83-4.51)
Remote VT					
10-30 y ago^d^	18	6	2.59 (1.00-6.67)	2.51 (0.90-6.97)	2.33 (0.81-6.67)
>30 y ago^d^	8	4	1.73 (0.51-5.83)	1.03 (0.27-3.96)	0.98 (0.24-3.95)
Men					
No prior VT	166	209	1 [Reference]	1 [Reference]	1 [Reference]
All remote VT	17	8	2.68 (1.13-6.35)	3.86 (1.36-10.97)	3.51 (1.19-10.35)
Remote VT					
10-30 y ago^d^	13	5	3.27 (1.14-9.37)	3.80 (1.20-11.99)	3.17 (0.96-10.47)
>30 y ago^d^	2	2	1.26 (0.18-9.03)	2.10 (0.14-30.74)	2.10 (0.11-40.52)
Women					
No prior	235	222	1 [Reference]	1 [Reference]	1 [Reference]
All remote VT	42	17	2.33 (1.29-4.22)	2.06 (1.10-3.86)	1.87 (0.96-3.64)
Remote VT					
10-30 y ago^d^	15	6	2.36 (0.90-6.20)	1.77 (0.64-4.94)	1.35 (0.45-4.02)
>30 y ago^d^	25	10	2.36 (1.11-5.03)	2.21 (1.00-4.93)	2.28 (0.98-5.31)
BMI <25^e^					
No prior VT	127	154	1 [Reference]	1 [Reference]	1 [Reference]
All remote VT	22	6	4.45 (1.75-11.30)	4.15 (1.57-10.97)	4.88 (1.64-14.56)
Remote VT					
10-30 y ago^d^	11	3	4.45 (1.21-16.28)	3.86 (0.98-15.23)	3.45 (0.82-14.51)
>30 y ago^d^	11	3	4.45 (1.21-16.28)	4.43 (1.15-17.02)	7.28 (1.36-39.06)
BMI ≥25^e^					
No prior VT	254	269	1 [Reference]	1 [Reference]	1 [Reference]
All remote VT	33	17	2.06 (1.12-3.78)	1.84 (0.96-3.52)	1.59 (0.81-3.14)
Remote VT					
10-30 y ago^d^	16	8	2.12 (0.89-5.04)	2.10 (0.84-5.29)	1.56 (0.58-4.16)
>30 y ago^d^	13	8	1.72 (0.70-4.22)	1.34 (0.52-3.45)	1.35 (0.51-3.54)

Abbreviations: BMI, body mass index (calculated as weight in kilograms divided by height in meters squared); VT, venous thrombosis.

^a^ Adjusted for age, sex, BMI, study center, and family history of VT (standard factors).

^b^ Adjusted for age, sex, BMI, study center, family history of VT, factor V Leiden, variant in the prothrombin gene (factor II A^20210^), and non–O blood groups (extensive factors).

^c^ Mean age of the total study population.

^d^ Exact time between former VT and index date is missing for 4 patients and 1 control participant.

^e^ Mean BMI in patients and control participants.

## Discussion

In this population aged 70 years and older, a remote history of VT was associated with an increased risk of VT independent of other factors, including common gene variants. Similar risk patterns were observed for VT types, albeit the relative risks were more pronounced for DVT than for PE with or without DVT, and more pronounced for provoked events than unprovoked events. Associations were robust in various important subgroups and similar for remote VT that occurred 20 to 30 years previous or 10 to 20 years previous. Because the OR was modest and the proportion of cases with a remote history of VT relatively low (12.8%), the PAR of a remote history of VT alone was only 7.7%. Nevertheless, findings highlight the importance of considering lifetime history of VT in older people.

Several cohort studies showed that a previous history of VT was associated with recurrence of VT in the population younger than 70 years and a previous history of VT was independently associated with VT recurrence.^9,10,11,12,13,14^ However, these studies generally had a short follow-up of less than 10 years, so evaluation of the association of prior VT with health and VT risk in older people could not be determined. The findings of the present study are important because they provide clinicians with quantitative information that might allow them to be aware of a future VT in people presenting for primary care or with impending provoking VT risk settings such as surgery. Consideration of risk models for VT in this age group requires further study, and such studies should consider remote as well as recent history of VT (for which there is more information).

### Strengths and Limitations

The main strength of our study is that, to our knowledege, it is one of the largest studies on VT risk in older adults. We were able to recruit older individuals by performing home visits and achieved a high participation rate. Furthermore, we had a sufficient number of individuals with a history of remote VT, enabling us to assess the association between this history and the risk of VT. We also had detailed information on other risk factors of VT, both standard available risk factors and genetic markers. This allowed us to further assess the adjusted association of a remote history of VT.

This study also had some limitations that may have led to underestimation of the true risk associated with remote history of VT. Because we use self-reported information on remote history of VT, we do not have data on VT characteristics or treatments for the remote VT; however, in the time frame of study, VT was usually treated with time-limited vitamin K antagonists. For 3 of the 84 remote VT events, it was unclear whether they had DVT/PE or only superficial VT. Excluding these individuals did not materially change the results. Self-report of remote VT may be affected by recall bias because patients may better recall a history of VT than control participants. However, for both patients and control participants, the time since a previous event was more than 10 years, so it is likely for both groups that it was equally difficult to remember. Thus, recall bias should not have major implications for observed risk estimates. Potentially, if older people in general have difficulties remembering distant events, nondifferential misclassification would only lead to underestimation of the risk.

Individuals who had a thrombotic event more than 10 years ago had to survive until at least age 70 years to be considered as participant in our study, which would lead to biases in estimating the strength for a study of VT cause. However, we did not aim to infer causality. The study participants were predominantly of White race and ethnicity, so results cannot be generalized to other races or ethnicities. The CIs for risk estimates in subgroups became wide, so differences between subgroups should be interpreted with caution. Owing to the case-control study design, absolute risk cannot be determined, but we were able to determine the risks of developing VT according to exposures of interest. It is likely that the risk of VT associated with remote VT of provoked VT was lower than that of unprovoked VT owing to the routine use of prophylaxis in high-risk settings, reducing provoked VT.

## Conclusions

In this case-control study, a remote history of VT was associated with an increased risk of VT at older age. Information on a remote history of VT may help to identify older people who are at increased risk of VT. To fully incorporate this finding into clinical practice, study of the added value of this factor to prediction models in this age group requires further investigation.
